# A Comparative Study: The Use of Collagen Implant versus Mitomycin-C in Combined Trabeculotomy and Trabeculectomy for Treatment of Primary Congenital Glaucoma

**DOI:** 10.1155/2017/9241459

**Published:** 2017-04-23

**Authors:** Alaa Abdel Sadek Singab, Osama Ali Mohammed, Mohammed Iqbal Hafez Saleem, Mortada Ahmed Abozaid

**Affiliations:** Ophthalmology Department, Sohag University, Sohag, Egypt

## Abstract

**Purpose:**

To compare Ologen implant versus mitomycin-C (MMC) in combined trabeculotomy and trabeculectomy as a treatment of primary congenital glaucoma.

**Setting:**

Sohag University Hospital, Egypt.

**Design:**

A prospective comparative study.

**Methods:**

Thirty-four eyes of twenty-one patients with primary congenital glaucoma were included in this study. All patients were subjected to preoperative evaluation including complete anterior segment examination under general anesthesia. The patients were divided into two groups: patients of the first group (group A) underwent combined trabeculotomy and trabeculectomy with Ologen implantation while those of the second group (group B) underwent combined trabeculotomy and trabeculectomy with MMC application.

**Results:**

Postoperatively, the IOP in group A was as follows: 8 eyes developed IOP levels less than 14 mmHg (complete success), 3 eyes had levels between 14 and 16 mmHg (accepted result), 2 eyes had levels between 16 and 20 mmHg (guarded result), and only 2 eyes showed levels exceeding 20 mmHg (failed procedure), while in group B, 7 eyes showed complete success, 3 eyes had accepted result, 3 eyes had guarded result, and 2 eyes had failed procedure.

**Conclusion:**

Ologen is a safe and effective adjuvant in combined trabeculotomy and trabeculectomy for treatment of primary congenital glaucoma.

## 1. Introduction

Congenital glaucoma (CG) is a major cause of blindness in children, despite its low incidence (1 : 10,000 births). It may be isolated (primary congenital glaucoma) or associated with other developmental anomalies, either systemic or ocular. The eyes with primary congenital glaucoma have an isolated maldevelopment of the trabecular meshwork not associated with other developmental ocular anomalies or ocular diseases that can raise intraocular pressure. It is the most common glaucoma of infancy, occurring in about 1 : 30,000 live births [1].

Primary congenital glaucoma (PCG) is a bilateral disease in about 75% of cases, with males accounting for approximately 65% of cases. Most cases are sporadic in occurrence, with no evident hereditary pattern. In approximately 10% of cases, a hereditary pattern is evident; it is believed to be autosomal recessive. Many authors believe that the inheritance pattern is polygenic [2].

Congenital glaucoma is essentially a surgical disease, in which surgery must be performed as early as possible. Goniotomy and trabeculotomy are usually the first procedures of choice. Both are safe and have a low incidence of complications. When goniotomies or trabeculotomies fail or are impossible, trabeculectomy is the usual alternative. Glaucoma drainage implants, nonpenetrating surgery, and cyclodestructive procedures are other options [3, 4].

Combined trabeculotomy and trabeculectomy is the most common procedure for congenital glaucoma in our locality because many cases present late with advanced disease. It allows high chance of success from the first intervention and reduces the need for secondary intervention which carries high failure rate.

MMC is a chemotherapeutic agent that has been widely used intraoperatively to enhance the success rate of glaucoma filtration surgery [5–8]. However, it is frequently accompanied with short- and long-term postoperative complications such as hypotony, bleb leaks, cataract formation, avascular filtering blebs, thinning of the conjunctiva, subsequent blebitis, choroidal effusions, maculopathy, and endophthalmitis [9]. Therefore, there is still a need for other adjuvants with similar or better efficacy and less complications.

Recently, several tissue-engineered biodegradable implants have been introduced to augment success of trabeculectomy with less complications than MMC [10].

Ologen (Aeon Astron Europe BV, Leiden, The Netherlands) is a biodegradable collagen-glycosaminoglycan (GAG) implant which decreases early postoperative scarring and prevents collapse of the subconjunctival space [11–13]. It is a disc-shaped porcine-derived collagen matrix that is inserted under the conjunctiva at the time of trabeculectomy, acting as a reservoir and helping to mechanically separate the conjunctiva from episcleral surface preventing adhesions between them. After implantation, the device should completely degrade within 90–180 days [14, 15].

This study aims to verify the safety and efficacy of Ologen implant compared to MMC application as an adjuvant in the surgical treatment of primary congenital glaucoma.

## 2. Patients and Methods

Thirty-four eyes of twenty-one patients with primary congenital glaucoma were included in this study. Thirteen patients underwent surgery in their both eyes and eight patients had surgery in one eye. Approval from ethical committee of Sohag Faculty of Medicine was obtained, and a written informed consent was obtained from the parents of all children after explaining the benefits and risks of the procedure.

Surgery was done by 4 surgeons to all infants and children with primary congenital glaucoma admitted to the Department of Ophthalmology in Sohag University Hospital in the period from April 2014 to October 2015.

The inclusion criteria of the study were patients aged less than three years with primary congenital glaucoma as evidenced by history of lacrimation, photophobia, blepharospasm, and/or eye enlargement in additition to the signs of elevated IOP, increased corneal diameters, corneal haze, and/or increased cup-to-disc ratio, while the exclusion criteria included patients with secondary glaucoma; patients with other ocular pathologies, for example, congenital cataract; patients with previous ocular surgery including glaucoma surgery; and patients who could not adhere to the follow-up schedule (lost from follow-up for more than two visits).

All patients were subjected to preoperative evaluation including history taking from the parents; the data collected included age, sex, main symptoms, family history, and medical history. The patients were referred for full systemic evaluation by a pediatrician to rule out any associated systemic anomalies. Under general anesthesia, complete anterior segment examination was done including measurement of the corneal diameters with surgical caliper (*vertical and horizontal*) and measurement of IOP using Perkin's applanation tonometer. Indirect fundus examination and refraction were done if the corneal clarity permits. Preoperative ocular ultrasonography was done for cases in which the fundus cannot be seen.

The patients were divided into two equal groups, each included 17 eyes (odd numbers for the first group and even numbers for the second group). In the first group (OLO group, group A), the patients had combined trabeculotomy and trabeculectomy with Ologen implantation while in the second group (MMC group, group B), the patients had combined trabeculotomy and trabeculectomy with MMC application.

## 3. Surgical Techniques

All surgeries were done under general anesthesia.

### 3.1. Group (A): Combined Trabeculotomy and Trabeculectomy with Collagen Matrix Implant (OLO Implant)

Antiseptic solution (povidone-iodine 7.5%) was applied to the periocular area, and a sterile ophthalmic surgical drape was applied.

The technique started with corneal traction suture including partial thickness at 12 o'clock using 6-0 silk and with creating a superior fornix-based conjunctival flap by dissection of the conjunctiva and Tenon's fascia to show bare sclera. Compression hemostasis was attained by cotton sponge compression or gentle diathermy if needed.

A 4 × 4 mm triangular limbal-based partial-thickness scleral flap was dissected extending into about 1 mm of the clear cornea leaving about two-thirds of the scleral thickness as a scleral bed. A radial incision was then carried out about 2 mm from the limbus at the junction between white and bluish zones of the sclera to enter the Schlemm's canal, evidenced by a gush of aqueous humour and/or blood, its characteristic pearly white color and appearance of transverse fibers running at the floor of the canal. Trabeculotomy was then performed using the internal arm of Harm's trabeculotome probes, first to the left and then to the right to complete about 100–120° of the circumference. Then, a 2 × 2 mm trabecular meshwork block was excised and a peripheral iridectomy was then performed.

A cylindrical collagen matrix implant (1 mm in height and 12 mm in diameter) was used. The implant was divided unequally into two parts: a smaller part and a larger part. The smaller part was implanted under the scleral flap over the scleral bed, and the scleral flap was closed with one 10-0 nylon suture leaving the two ends of the smaller part bulging from both sides of the scleral flap. The remaining larger part was inserted in the sub-Tenon's space over the scleral flap, and then, a watertight closure of the conjunctival flap was done with continuous buried 10.0 nylon stitches.

### 3.2. Group (B): Combined Trabeculotomy and Trabeculectomy with MMC Application

The technique was similar to that of the first group except that four sponges soaked with MMC with a concentration of 0.4 mg/ml were placed deeply in the subconjunctival space as follows: one sponge at 12 o'clock, two sponges on both sides of superior rectus position, and the fourth sponge was applied between the scleral bed and the scleral flap and left for 2 minutes followed by irrigation of the eye with balanced salt solution. The scleral flap was closed with one tight 10-0 nylon suture at its apex and two 10-0 nylon sutures at both sides of the scleral flap.

The postoperative treatment of all patients included moxifloxacin 0.5% eye drops 5 times daily for 2 weeks and prednisolone acetate 1% eye drops five times daily for one week, three times daily for another week, and once daily for a final week, as well as cyclopentolate eye drops twice daily for one week.

## 4. Schedule of Follow-Up Visits

All patients were seen daily for the first few days looking for eye injection, corneal edema, anterior chamber formation, and red reflex; then, postoperative visits were scheduled at one week; two weeks; and one, two, four, six, nine, and twelve months. Patient examination was performed under general anesthesia for IOP, corneal clarity and diameters, bleb status, fundus examination, and postoperative complications.

## 5. Statistical Analysis

Data was analyzed using SPSS computer program version 16.0. (SPSS Inc, Chicago, Intl). The data were tested for normality using Shapiro-Wilk test which was insignificant indicating the use of parametric tests as data was normally distributed. Quantitative data were expressed as means ± standard deviation and were analyzed by using *t*-tests and paired samples *t*-test. Independent samples *t*-test was used to assess statistical significance between both groups in predetermined parameters. While paired samples *t*-test was used to assess statistical significance within the same group. Chi-square (*χ*^2^) test and Fisher's exact tests were used for comparison regarding qualitative variables. A 5% level was chosen as a level of significance in all statistical tests used in the study.

## 6. Results

This study included thirty-four eyes of twenty-one patients having primary congenital glaucoma. Thirteen patients had surgery in both eyes and eight patients in only one eye. These eyes were divided equally into two groups: seventeen eyes for the OLO group (group A) and seventeen eyes for the MMC group (group B). Three eyes did not complete the follow-up schedule and lost from follow-up for more than two visits, so they were excluded from the study. In addition, intraoperative inadvertent scleral perforation occurred in one case, and thus, such case was excluded from the study. Finally, thirty eyes of eighteen patients fulfilled the inclusion criteria. Fifteen eyes of nine patients (six bilateral and three unilateral) were included in each group for this study.

The preoperative characteristics did not differ significantly between the 2 groups. In group A, there were 7 (77%) boys and 2 (23%) girls while in group B, 6 (66%) boys and 3 (33%) girls. The mean age was 9 ± 4 months in group A and 8 ± 5 months in group B with a *P* value of 1.00.

The mean preoperative IOP was 30.5 ± 2.6 mmHg in group A and 31.1 ± 3.3 mmHg in group B with a *P* value of 0.50. The mean TCD was 12.5 ± 0.50 mm in group A and 13 ± 1 mm in group B with a *P* value of 0.21. Corneal edema was present in 13 eyes (86.7%) of group A and in 11 eyes (73.3%) of group B.

## 7. Postoperative Results

### 7.1. The Intraocular Pressure

According to the level of IOP, the patients in each group were included in one of the four subgroups as follows:

- Subgroup I: IOP range was less than or equal to 14 mmHg. The surgery was successful especially if associated with improvement of symptoms, stable transverse corneal diameter, and improvement of corneal haze and edema. Cases complicated with early bleb leakage and hypotony were excluded from this subgroup.
- Subgroup II: IOP range was between 14 and 16 mmHg reflecting accepted results with remote possibility of additional surgical procedures in the future.
- Subgroup III: IOP level was between 16 and 20 mmHg reflecting guarded results with the possibility of the need for antiglaucoma medication or even additional surgical procedures in the future specially if there was persistence of symptoms or corneal edema.
- Subgroup IV: IOP level exceeded 20 mmHg reflecting unsuccessful procedure with the need for another surgical procedure to control the IOP.

## 8. Group A (OLO Group)

The mean preoperative IOP was 30.5 mmHg, and the mean postoperative IOP at the end of the study was 15.4 mmHg with a *P* value of 0.00 indicating a highly significant reduction in the mean IOP level. The mean reduction of IOP was 15.1 mmHg.

One week postoperatively, 12 eyes (80%) had IOP levels less than or equal to 14 mmHg; one eye showed IOP level between 14 and 16 mmHg while the remaining two eyes had IOP levels between 16 and 20 mmHg.

By the end of the study (after one year), 8 eyes (53.3%) developed IOP levels less than 14 mmHg, that is, complete success; three eyes (20%) had IOP levels ranging between 14 and 16 mmHg, that is, accepted result; two eyes (13.3%) had IOP levels ranging between 16 and 20 mmHg, that is, guarded result; and only two eyes (13.3%) showed IOP levels exceeding 20 mmHg, that is, failed procedure.

## 9. Group B (MMC Group)

The mean preoperative IOP was 31.1 mmHg, and the mean IOP at the end of the study was 17.3 mmHg with a *P* value of 0.00 indicating a highly significant reduction in IOP level. The mean reduction of IOP was 13.9 mmHg.

One week postoperatively, 14 eyes (93.3%) had IOP levels less than or equal to 14 mmHg; one eye (6.7%) showed IOP level between 16 and 20 mmHg.

By the end of the study, 7 eyes (46.7%) developed IOP levels less than 14 mmHg, that is, complete success; three eyes (20%) had IOP levels ranging between 14 and 16 mmHg, that is, accepted result; three eyes (20%) had IOP levels ranging between 16 and 20 mmHg, that is, guarded result; and only two eyes (13.3%) showed IOP levels exceeding 20 mmHg, that is, failed procedure.

During the whole follow-up period, there was statistically insignificant difference between both groups with *P* values of 0.399, 0.35, 0.85, 0.523, 0.511, 0.323, 0.263, and 0.471 for follow-up visits (Table 1).

## 10. Corneal Diameters

The mean TCD was 12.5 ± 0.50 mm in group A and 13 ± 1 mm in group B with a *P* value of 0.21 while the average postoperative TCD at the end of the study was 13.01 mm in group A and 13.34 mm in group B (*P* value = 0.466; statistically insignificant). In group A, TCD was stable in 8 eyes (53%) and increased by average 0–0.5 mm in 7 eyes (47%). In group B, TCD was stable only in 5 eyes (33%) and increased by 0.5–1 mm in 10 eyes (66%).

## 11. Corneal Clarity

Corneal edema was present in 13 eyes (86.7%) of group A and in 11 eyes (73.3%) of group B. By the end of the follow-up period, 9 eyes (60%) of group A and 7 eyes (46.7%) of group B showed a clear cornea. 5 eyes (33.3%) of group A and 6 eyes (40%) of group B showed persistent corneal haze. Only one eye (6.7%) in group A and two eyes (13.3%) in group B developed permanent corneal opacification.

## 12. Bleb Evaluation

All cases of both groups were subjected to surgery at 12 o'clock, so in all cases, the bleb was present superiorly and the bleb height was evaluated under a surgical microscope under general anesthesia (Table 2).

Bleb vascularity is shown in Table 3.

## 13. Postoperative Complications

The frequency of postoperative complications did not significantly differ between the two groups (Table 4).

No allergic reaction to the OLO, matrix extrusion, or conjunctival erosion was noted in the OLO group. No cases of endophthalmitis were encountered in both groups. Only 2 cases (13.3%) in the OLO group necessitated the use of antiglaucoma medications during the follow-up period in the form of B-blockers and dorzolamide to control IOP level and other parameters. While in MMC, similar medications were to be prescribed in 4 cases (26.7%).

In the OLO group, only one case (6.7%) with IOP exceeding 21 mmHg failed to be controlled on two antiglaucoma medications necessitated a secondary surgical intervention in the form of combined procedure using MMC. Unfortunately, the second intervention also failed to halt the progression, and the case is still under follow-up waiting for glaucoma shunt surgery.

On the other hand, two cases (13.3%) in the MMC group necessitated a secondary surgical intervention in the form of combined procedure using MMC. The average IOP level reached 17 mmHg in one case and 19 mmHg in the other case with the use of antiglaucoma medication.

Regarding hyphema (two eyes in each group), it improved conservatively by mydriatics and steroids in all cases within 7 days.

In MMC, one of 3 cases complicated with hypotony and early bleb leakage was managed conservatively by mydriatics and withdrawal of steroids, while the other two cases necessitated surgical intervention in the form of tight resuturing of the conjunctival flap to prevent bleb leakage.

No cases of hypotony or early bleb leakage were encountered in the OLO group. Three cases encountered choroidal detachment (one eye in the OLO group and two eyes in the MMC group). All cases were managed conservatively by mydriatics and steroids.

Regarding corneal scarring, only one eye (6.7%) had postoperative scarring in the OLO group while two eyes (13.3%) in the MMC group.

## 14. Discussion

Congenital glaucoma is a major cause of blindness in children, despite its low incidence (1 : 10,000 births) [1]. In this study, a comparison between Ologen (group A) and MMC (group B) application in combined trabeculotomy and trabeculectomy for treatment of primary congenital glaucoma was done to verify efficacy and safety of Ologen.

As regards preoperative characteristics of the patients, there was no significant difference between the 2 groups. Postoperatively, the two groups showed a highly significant reduction (*P* value < 0.01) in IOP levels.

This significant reduction in both groups can be explained by the surgical procedure which was combined trabeculotomy and trabeculectomy. However, this level of reduction in the mean IOP cannot be relied on as an indicator of success due to presence of cases with very high preoperative IOP on the one hand and cases complicated with bleb leakage and hypotony on the other hand.

Although the frequency of postoperative complications especially hypotony was higher in the MMC group than the OLO group, the difference does not reach statistical significance probably due to the small sample size of the study.

Ologen implant could have a tamponading effect and provide a controlled drainage of aqueous. In addition, the implant might have a valve-like mechanism through its two parts: subscleral and subconjunctival parts; when they were inflated with aqueous, more pressure on the sclera flap would be exerted decreasing aqueous flow and vice versa.

After its success in animals [16, 17], many studies tried to verify the efficacy and safety of Ologen in trabeculectomy for primary open-angle glaucoma and other glaucomas of adults [18–22]. However, few studies verified it in primary congenital glaucoma.

In his pilot study, Hamdi [23] used subconjunctival Ologen implant as an adjuvant to subscleral trabeculectomy in 3 cases of primary congenital glaucoma. After a follow-up of 6–8 months, results were as follows: “satisfactory success” for the first case, “full success” for the second case, and “poor success” for the third case. He reported that the advantages of this implant are its safety over MMC and its ease of use. Ologen would prevent secondary interference such as needling after the use of antifibrotic agents and also would avoid tube-related complications.

Unlike Hamdi, we used Ologen both subconjunctivally and subsclerally to augment effects of combined trabeculotomy and trabeculectomy and avoiding risks of MMC and we followed up cases for one year.

In his study over twenty eyes of 15 patients with primary congenital glaucoma, Hafez [24] divided cases into two groups. The first group (MMC group) included 10 eyes and was subjected to trabeculectomy with MMC. The second group (OLO group) included 10 eyes and was subjected to trabeculectomy with a collagen matrix implant (Ologen). The postoperative IOP level was classified into four groups (>21, >17–21, 15–17, and <15 mmHg). At the end of the sixth postoperative follow-up month, in the MMC group, only 10% of eyes achieved the target IOP, 10% of eyes had failed surgery, and 80% of eyes had IOP ranging from 15 to 21 mmHg. However, in the Ologen group, 40% of eyes achieved the target IOP, 60% of eyes had IOP ranging from 15 to 21 mmHg, and there were no failed surgeries. In terms of complications, the MMC group had a higher rate of complications than the Ologen group in the form of early hyphema, bleb leakage, hypotony, and choroidal detachment.

Unlike Hafez, we used Ologen with combined trabeculotomy and trabeculectomy and we followed up cases for one year.

Elmallah et al. [25] compared trabeculotomy with deroofing of Schlemm's canal augmented with subconjunctival Ologen implant (study group) and conventional trabeculotomy (control group) in primary congenital glaucoma and found that the success rate was higher in the study group (100%) than in the control group (75%) after a 6 month follow-up period.

Unlike Elmallah et al., this study used Ologen (both subscleral and subconjunctival) with combined trabeculotomy and trabeculectomy and cases were followed up for one year with a success rate of about 87%.

The major limitations of this study are the small sample size and the relatively short follow-up period. Future studies should be carried out on larger samples and for longer follow-up periods to establish the long-term safety and efficacy of this new device. Ologen implant can be also tried in cases with secondary glaucoma, resistant glaucoma, and congenital glaucoma associated with other ocular congenital anomalies.

In conclusion, this study suggests that the collagen matrix implant (Ologen) is a safe and effective adjuvant in combined trabeculotomy and trabeculectomy for treatment of primary congenital glaucoma. Although there was insignificant difference between Ologen and MMC as regards efficacy, Ologen appears to be safer than MMC regarding postoperative complications especially postoperative hypotony.

## Figures and Tables

**Table 1:** Results of IOP during the follow-up period in both groups.

Follow-up visits	Group A (OLO)				Group B (MMC)			
	Subgroup (I)	Subgroup (II)	Subgroup (III)	Subgroup (IV)	Subgroup (I)	Subgroup (II)	Subgroup (III)	Subgroup (IV)	*P* value
1st week	12 (80%)	1 (6.7%)	2 (13.3%)	0 (0.0%)	14 (93.3%)	0 (0.0%)	1 (6.7%)	0 (0.0%)	0.399
2nd week	12 (80%)	1 (6.7%)	2 (13.3%)	0 (0.0%)	13 (86.7%)	1 (6.7%)	1 (6.7%)	0 (0.0%)	0.35
1st month	12 (80%)	1 (6.7%)	2 (13.3%)	0 (0.0%)	12 (80%)	2 (13.3%)	0 (0.0%)	1 (6.7%)	0.85
2nd month	10 (66.7%)	3 (20%)	1 (6.7%)	1 (6.7%)	8 (53.3%)	3 (20%)	1 (6.7%)	1 (6.7%)	0.523
4th month	8 (53.3%)	4 (26.7%)	2 (13.3%)	1 (6.7%)	9 (60%)	4 (26.7%)	1 (6.7%)	1 (6.7%)	0.511
6th month	9 (60%)	2 (13.3%)	3 (20%)	1 (6.7%)	5 (33.3%)	5 (33.3%)	4 (26.7%)	1 (6.7%)	0.323
9th month	8 (53.3%)	4 (26.7%)	2 (13.3%)	1 (6.7%)	5 (33.3%)	4 (26.7%)	4 (26.7%)	2 (13.3%)	0.263
12th month	8 (53.3%)	3 (20%)	2 (13.3%)	2 (13.3%)	7 (46.7%)	3 (20%)	3 (20%)	2 (13.3%)	0.471

**Table 2:** Bleb height during follow-up in both groups.

	OLO group			MMC group		
	1st month	At 6th month	At 12th month	1st month	At 6th month	At 12th month
Flat	1 (6.7%)	3 (60%)	4 (26.7%)	9 (60%)	6 (40%)	7 (46.7%)
Moderate elevation	3 (20%)	5 (33.3%)	6 (40%)	4 (26.7%)	5 (33.3%)	4 (26.7%)
Well formed	11 (73.3%)	7 (46.7%)	5 (33.3%)	2 (13.3%)	4 (26.7%)	4 (26.7%)

**Table 3:** Bleb vascularity during follow-up in both groups.

	OLO group			MMC group		
	1st month	At 6th month	At 12th month	1st month	At 6th month	At 12th month
Avascular	0 (0.00%)	2 (13.3%)	4 (26.7%)	1 (6.7%)	3 (20%)	5 (33.3%)
Normal vascularization	10 (66.7%)	11 (73.3%)	10 (66.7%)	11 (73.3%)	9 (60%)	8 (53.3%)
Vascular inflammation	5 (33.3%)	2 (13.3%)	1 (6.7%)	3 (20%)	3 (20%)	2 (13.3%)

**Table 4:** Frequency of postoperative complications in both groups.

Complication	Hypotony	Corneal scarring	Hyphema	Choroidal detachment	Failed procedure
OLO group	0 (0.0%)	1 (6.7%)	2 (13.3%)	1 (6.7%)	2 (13.3%)
MMC group	3 (20%)	2 (13.3%)	2 (13.3%)	2 (13.3%)	2 (13.3%)
*P* value	0.072	0.559	1	0.559	1

## References

[1] Dickens C. J., Hoskins J. R. H. D., Ritch R., Shields M. B., Krupin T. (1996). Epidemiology and pathophysiology of congenital glaucoma. *The Glaucomas*.

[2] Mandal A. K., Netland P. A. (2006). Primary congenital glaucoma. *The Pediatric Glaucomas*.

[3] Dickens C. J., Hoskins J. R. H. D., Ritch R., Shields M. B., Krupin T. (1996). Diagnosis and treatment of congenital glaucoma. *The Glaucomas*.

[4] Essuman V. A., Braimah I. Z., Ndanu T. A., Ntim-Amponsah C. T. (2011). Combined trabeculotomy and trabeculectomy: outcome for primary congenital glaucoma in a West African population. *Eye (London, England)*.

[5] Waheed S., Ritterband D. C., Greenfield D. S., Liebmann J. M., Sidoti P. A., Ritch R. (1997). Bleb-related ocular infection in children after trabeculectomy with mitomycin C. *Ophthalmology*.

[6] Lin Z. J., Li Y., Cheng J. W., Lu X. H. (2012). Intraoperative mitomycin C versus intraoperative 5-fluorouracil for trabeculectomy: a systematic review and meta-analysis. *Journal of Ocular Pharmacology and Therapeutics*.

[7] Hollo G. (2012). Wound healing and glaucoma surgery: modulating the scarring process with conventional antimetabolites and new molecules. *Developments in Ophthalmology*.

[8] Cheng J. W., Cai J. P., Li Y., Wei R. L. (2011). Intraoperative mitomycin C for nonpenetrating glaucoma surgery: a systematic review and meta-analysis. *Journal of Glaucoma*.

[9] Cheng J. W., Xi G. L., Wei R. L., Cai J. P., Li Y. (2009). Efficacy and tolerability of nonpenetrating glaucoma surgery augmented with mitomycin C in treatment of open-angle glaucoma: a meta-analysis. *Canadian Journal of Ophthalmology*.

[10] Seibold L. K., Sherwood M. B., Kahook M. Y. (2012). Wound modulation after filtration surgery. *Survey of Ophthalmology*.

[11] Rao K., Ahmed I., Blake D. A., Ayyala R. S. (2009). New devices in glaucoma surgery. *Expert Review of Ophthalmology*.

[12] Zelefsky J. R., Hsu W. C., Ritch R. (2008). Biodegradable collagen matrix implant for trabeculectomy. *Expert Review of Ophthalmology*.

[13] Nilforushan N., Yadgari M., Falavarjani K. G. (2010). Evaluation of subconjunctival Ologen implantation as an adjunct to trabeculectomy. *Iranian Journal of Ophthalmology*.

[14] Rosentreter A., Mellein A. C., Konen W. W., Dietlein T. S. (2010). Capsule excision and Ologen implantation for revision after glaucoma drainage device surgery. *Graefe's Archive for Clinical and Experimental Ophthalmology*.

[15] Senthil S., Rao H. L., Babu J. G., Mandal A. K., Garudadri C. S. (2013). Comparison of outcomes of trabeculectomy with mitomycin C vs. ologen implant in primary glaucoma. *Indian Journal of Ophthalmology*.

[16] Chen H. S., Ritch R., Krupin T., Hsu W. C. (2006). Control of filtering bleb structure through tissue bioengineering: an animal model. *Investigative Ophthalmology & Visual Science*.

[17] Hsu W. C., Ritch R., Krupin T. (2008). Tissue bioengineering for surgical bleb defects: an animal study. *Graefe's Archive for Clinical and Experimental Ophthalmology*.

[18] Shaarawy T., Nguyen C., Schnyder C., Mermoud A. (2004). Comparative study between deep sclerectomy with and without collagen implant: long term follow up. *The British Journal of Ophthalmology*.

[19] Shaarawy T., Mermoud A. (2005). Deep sclerectomy in one eye vs deep sclerectomy with collagen implant in the contralateral eye of the same patient: long-term follow-up. *Eye (London, England)*.

[20] Boey P. Y., Narayanaswamy A., Zheng C. (2011). Imaging of blebs after phacotrabeculectomy with Ologen collagen matrix implants. *The British Journal of Ophthalmology*.

[21] Papaconstantinou D., Georgalas I., Karmiris E. (2010). Trabeculectomy with Ologen versus trabeculectomy for the treatment of glaucoma: a pilot study. *Acta Ophthalmologica*.

[22] Rosentreter A., Schild A. M., Jordan J. F., Krieglstein G. K., Dietlein T. S. (2010). A prospective randomised trial of trabeculectomy using mitomycin C vs an ologen implant in open angle glaucoma. *Eye (London, England)*.

[23] Hamdi M. M. (2013). Trabeculectomy assisted by collagen matrix implant (Ologen) in primary congenital glaucoma. *Journal Egypt Ophthalmol soc*.

[24] Hafez M. I. (2014). Trabeculectomy with collagen matrix implantation versus trabeculectomy with mitomycin C application for treatment of primary congenital glaucoma. *Highlights of Ophthalmology.*.

[25] Elmallaha M. A. H., Mohamed T. H., Elmahdya A. G. (2016). A comparative study between trabeculotomy with deroofing of Schlemm’s canal augmented with Ologen implant and conventional trabeculotomy in primary congenital glaucoma. *Delta Journal of Ophthalmology*.

